# Associations between different triglyceride glucose index-related obesity indices and periodontitis: results from NHANES 2009–2014

**DOI:** 10.1186/s12944-024-02192-z

**Published:** 2024-07-05

**Authors:** Liyuan Yang, Shiyan Fang, Runzhen Zhang, Rong Xia

**Affiliations:** Department of Stomatology, the Second Affiliated Hospital of Anhui Medical University, Hefei, 230601 China

**Keywords:** TyG index, TyG-related obesity index, Insulin resistance, Periodontitis, NHANES

## Abstract

**Background:**

This study aimed to explore the associations between triglyceride glucose (TyG) index-related obesity indices and periodontitis within the American population.

**Methods:**

This cross-sectional investigation utilized data from the National Health and Nutrition Examination Survey (NHANES) for 2009–2014. The association between the TyG–waist-to-height ratio (TyG-WHtR), TyG–weight-adjusted-waist index (TyG-WWI), TyG–waist circumference (TyG-WC), or TyG–body mass index (TyG-BMI) and periodontitis was investigated utilizing multivariable logistic regression model, subgroup, and dose-response curve analyses.

**Results:**

This study enrolled 4,808 adult participants. Except for TyG-BMI, which did not exhibit a relationship with periodontitis, TyG-WHtR, [odds ratio (OR) (95% confidence interval (CI))] = 2.83 [1.58–5.10], *P* = 0.002], TyG-WWI [OR (95% CI) = 7.50 (3.06–18.34), *P* < 0.001], and TyG-WC [OR (95% CI) = 2.12 (1.23–3.64), *P* = 0.011] were all associated with periodontitis. Participants in the highest quartile displayed an elevated risk of periodontitis relative to their counterparts in the lowest quartile, as evidenced for TyG-WWI [OR (95% CI) = 1.72 (1.26–2.33), *P* = 0.001] and TyG-WC [OR (95% CI) = 1.50 (1.13–1.99), *P* = 0.009] in the full adjustment model. Subgroup analyses suggested more pronounced positive associations between these indices and periodontitis in participants who were < 60 years old, had a BMI ≥ 25, and did not have diabetes. The dose-response curve indicated linear responses in these associations.

**Conclusions:**

This investigation identified a significant and stable association between TyG-WHtR, TyG-WWI, or TyG-WC and periodontitis, which implies a robust correlation between high insulin resistance and susceptibility to periodontitis in the American population.

**Supplementary Information:**

The online version contains supplementary material available at 10.1186/s12944-024-02192-z.

## Background

Periodontitis, a chronic inflammatory condition characterised by the progressive destruction of periodontal support tissues, exhibits a prevalence of up to 42% in adults in the United States and 11.2% globally for severe cases [1], making it the sixth most widespread human disease [2]. Periodontitis has been one of the primary contributors to tooth loss in adults [3], which not only impacts nutrition and quality of life but also aggravates global disease burden [4].

Remarkably, the systemic impact of periodontitis is of greater concern than its localized effects on oral health. For instance, periodontitis is related to an increased risk of metabolic syndrome, diabetes, and other chronic diseases [5–7]. Insulin resistance (IR) may assume a dominant part in this relationship. Thouvenot et al. [8] revealed that obesity promotes inflammatory factor secretion and inhibits insulin sensitivity, further exacerbating periodontal dysbiosis. Therefore, an extensive understanding of the link between obesity-related IR and periodontitis is essential in disease management.

Given the complexity of traditional IR index measurements, simpler assessment tools have been developed, such as the triglyceride glucose (TyG) index, which estimates insulin sensitivity from triglyceride (TG) and fasting glucose levels, and shows considerable clinical potential [9, 10]. Meanwhile, based on TyG, several new indices have been proposed combining obesity-related anthropological parameters to more accurately assess the severity of IR. For instance, the body mass index (BMI) is a widely accepted and standard tool for evaluating overall obesity and general health, and waist-to-height ratio (WHtR) is commonly utilized as an index for assessing abdominal obesity and its associated metabolic risks, as is the waist circumference (WC) [11, 12]. Furthermore, the weight-adjusted-waist index (WWI), a novel anthropometric measurement, has been suggested to be a superior index of central obesity compared to traditional BMI [13]. Given the significant correlation between an elevated TyG index and heightened risk of periodontitis, it was hypothesised that a novel composite index, incorporating TyG and obesity indices, may serve as a promising index for estimating periodontitis risk in individuals with IR. Notably, the composite metrics TyG-WHtR, TyG-WC, and TyG-BMI have shown a strong correlation with metabolic syndrome, diabetes, and other diseases and more accurately reflected diseases risk compared to TyG alone [14–16]. Furthermore, integrating TyG with obesity indices provides a more comprehensive assessment of the influence of IR severity resulting from diverse fat distributions on periodontitis. Nevertheless, no studies have assessed the correlations between these indices and periodontitis in the American population, which underscores the essential need for further confirmation of their correlations.

Therefore, by surveying the National Health and Nutrition Examination Survey (NHANES) database, the study analysed different TyG-related indices to explore the association between obesity-related IR and periodontitis risk.

## Methods

### Study design and participant selection

**Fig. 1:**
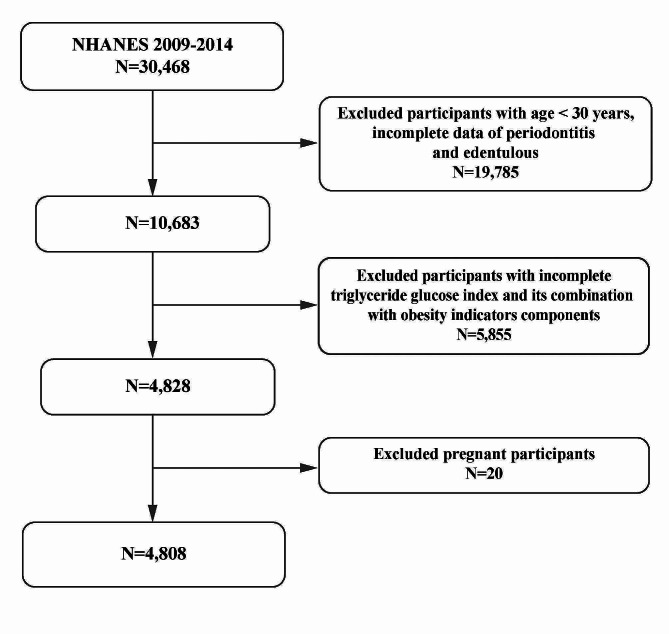
Flowchart of participants in this study

This study extracted data of 30,468 individuals from the NHANES database for the period 2009–2014. The NHANES utilizes a multistage stratified probability sampling strategy to collect extensive data on the health, lifestyle, and nutrition of the US citizens [17]. The full dataset comprised three pooled NHANES cycles for 2009–2010, 2011–2012, and 2013–2014, and strictly adhered to the following exclusion criteria: (1) participants aged < 30 years; (2) individuals with missing data on periodontitis; (3) edentulous adults; (4) incomplete data on TyG index (5) incomplete data on body measurement parameters; and (6) pregnant women. Ultimately, the study enrolled a total of 4,808 participants. The general data selection process is outlined in Fig. 1.

### Definitions and calculations of TyG-related obesity indices

The exposure variables TyG-WHtR, TyG-WWI, TyG-WC, and TyG-BMI were derived from the analysis of fasting glucose, TG, and certain body parameters. Baseline blood samples were gathered to measure fasting glucose and TG levels while anthropometric measurements were obtained by healthcare professionals with specialized training. The TyG index was calculated as TyG = ln [(TG levels × fasting glucose levels) / 2]. This index was then used to calculate the combined indices as previously described [18]: (1) WHtR = WC / Height; (2) $WWI=\frac{WC}{\sqrt{Weight}}$ ; (3) TyG-WHtR = TyG × WHtR; (4) TyG-WWI = TyG × WWI; (5) TyG-WC = TyG × WC; (6) TyG-BMI = TyG × BMI.

### Clinical periodontal assessment and definition

A comprehensive dental examination (excluding the third molars) was conducted by trained professionals. Measurements were performed at six sites for each tooth. The diagnostic assessment of the periodontal condition strictly adhered to the Centers for Disease Control and Prevention and American Academy of Periodontics definitions for the clinical attachment loss and probing pocket depth values at the four interproximal sites of each tooth [19]. Following a series of assessments, individuals diagnosed with mild, moderate, or severe periodontitis were assigned to the periodontitis group, whereas those without periodontitis were assigned to the non-periodontitis group.

### Assessment of covariates

A number of factors were incorporated as covariates, including gender, race, age, physical activity, educational level, BMI, alcohol consumption, smoking, income-to-poverty ratio (PIR), diabetes mellitus, hypertension, dental flossing frequency and dentition status. Gender was specifically classified as male or female. Age was divided into two groups with a threshold of 60. The PIR was grouped into two levels: “<1” and “≥1”. A BMI “<25” was considered within the normal weight range, while a BMI “≥25” was defined as overweight/obese. Alcohol consumption and smoking status were categorized as yes/no. Diabetes diagnosis was based on HbA1c levels of ≥ 6.5%, ≥ 126 mg/dL for fasting glucose level, ≥ 200 mg/dL for plasma glucose concentrations after an oral glucose tolerance test, physician confirmation, or self-reported use of insulin or diabetes drugs. Confirmation of a hypertension diagnosis occurred when any of the following criteria were satisfied: systolic blood pressure ≥ 40 mmHg (average of three consecutive readings), diastolic blood pressure ≥ 90 mmHg (average of three consecutive measurements), or individuals reporting a history of hypertension or utilizing antihypertensive medications. If dental floss was not used on any day during a week, the frequency of dental floss usage was marked as ‘No’ [20]. The assessment of dentition status depended on the presence of functional teeth: having 20 or more natural permanent teeth was considered functional dentition, whereas 19 or fewer indicated non-functional dentition [21].

### Statistical analyses

Considering the sophisticated multistage probability sampling design adopted in the NHANES, attention was given to the potential effects of sample weights, stratification, and clustering in the dataset. Consequently, the study grouped the TyG-WHtR, TyG-WWI, TyG-WC, and TyG-BMI estimates into quartiles and applied a weighted multivariable logistic regression model to analyse the associations of the TyG-related obesity indices with periodontitis. To ensure a comprehensive analysis, three adjustment models were formulated. Model 1 remained unadjusted; model 2 was subjected to adjustments for the demographic variables of age, gender, and race; and model 3 was adjusted for all potential confounders: age, gender, race, education level, PIR, BMI, physical activity, alcohol, smoking, hypertension, diabetes, dental floss and dentition status.

Given the right-skewed distribution of the data, a natural logarithm (ln) transformation was employed for continuous variables. Additionally, subgroup analyses based on the full adjustment model was applied to observe the associations between the TyG-related obesity indices and periodontitis in distinct subgroups of BMI, age, gender hypertension, and diabetes. The interaction test results were further analysed to identify the heterogeneity in the relationships within each subgroup. Finally, dose-response curves were used to identify possible connections between TyG-related obesity indices and periodontitis in the three models. The execution of statistical analyses involved R Studio (version 4.3.1) and Empower Stats (version 4.1), and statistical significance was set at *P* < 0.05.

## Results

### Baseline features of the study cohort

The essential traits of the participant characteristics are outlined in Table 1. The 4,808 individuals in the cohort showed an overall periodontitis prevalence of 50.12%. Participants aged < 60 years accounted for 73.3%, while those aged ≥ 60 years accounted for 26.7%. The cohort comprised 49.6% males and 50.4% females in gender distribution. As for race, the cohort included 8.1% Mexican Americans, 69.5% non-Hispanic whites, 9.7% non-Hispanic blacks, 5.6% with other Hispanic backgrounds, and 7.0% of other ethnicities.

**Table 1:** Baseline characteristics of the study population

	Overall	Non-Periodontitis	Periodontitis	*P* value
	(*N* = 4808)	(*N* = 2398)	(*N* = 2410)	
**Age (years)**	50.9 ± 0.3	48.2 ± 0.4	54.7 ± 0.4	< 0.001
**Age (%)**				< 0.001
< 60	73.3	79.7	64.2	
≥ 60	26.7	20.3	35.8	
**Gender (%)**				< 0.001
Male	49.6	43.7	57.9	
Female	50.4	56.3	42.1	
**Race (%)**				< 0.001
Mexican American	8.1	5.7	11.6	
Other Hispanic	5.6	5.0	6.5	
Non-Hispanic White	69.5	75.5	61.1	
Non- Hispanic Black	9.7	7.3	13.1	
Other Races	7.0	6.4	7.8	
**Education level (%)**				< 0.001
Less than high school	16.0	9.5	25.3	
High school and above	84.0	90.5	74.7	
**PIR (%)**				< 0.001
< 1	11.2	7.5	16.4	
≥ 1	88.8	92.5	83.6	
**BMI (%)**				0.422
< 25	26.7	27.3	25.9	
≥ 25	73.3	72.7	74.1	
**Alcohol intake (%)**				0.250
No	19.6	18.8	20.8	
Yes	80.4	81.2	79.2	
**Smoking (%)**				< 0.001
No	57.1	64.2	47.0	
Yes	42.9	35.8	53.0	
**Physical activity**				0.112
No	61.4	62.4	60.0	
Yes	38.6	37.6	40.0	
**Diabetes (%)**				< 0.001
No	83.5	88.3	76.6	
Yes	16.5	11.7	23.4	
**Hypertension (%)**				< 0.001
No	60.0	65.7	51.9	
Yes	40.0	34.3	48.1	
**Dental floss (%)**				< 0.001
No	28.5	23.8	35.1	
Yes	71.5	76.2	64.9	
**Dentition status (%)**				< 0.001
Non-functional	14.0	6.6	24.3	
Functional	86.0	93.4	75.7	
**TyG-WHtR**	5.15 ± 0.03	5.05 ± 0.03	5.30 ± 0.04	< 0.001
**TyG-WWI**	95.25 ± 0.29	93.56 ± 0.28	97.65 ± 0.48	< 0.001
**TyG-WC**	870.56 ± 4.26	853.69 ± 4.10	894.41 ± 6.63	< 0.001
**TyG-BMI**	253.83 ± 1.70	250.38 ± 1.82	258.70 ± 2.46	0.003
**TyG**	8.64 ± 0.02	8.57 ± 0.02	8.73 ± 0.03	< 0.001
**HOMA-IR**	3.69 ± 0.11	3.24 ± 0.11	4.32 ± 0.18	< 0.001
**Triglyceride (mg/dL)**	131.54 ± 2.58	125.25 ± 2.31	140.44 ± 4.92	0.006
**Fasting glucose (mg/dL)**	106.20 ± 0.70	102.38 ± 0.69	111.62 ± 1.02	< 0.001
**Fasting insulin (uU/mL)**	13.12 ± 0.29	12.18 ± 0.32	14.44 ± 0.45	< 0.001
**Waist circumference (cm)**	100.32 ± 0.33	99.16 ± 0.38	101.96 ± 0.49	< 0.001

Continuous variables were listed as Mean ± Standard deviation (SD), the *P*-value was derived using a weighted linear regression model

Categorical variables were listed as %, the *P*-value was derived using a weighted chi-square test

Abbreviations: PIR, income-to-poverty ratio; BMI, body mass index; TyG-WHtR, triglyceride glucose-waist to height ratio; TyG-WWI, triglyceride glucose-weight-adjusted-waist index; TyG-WC, triglyceride glucose-waist circumference; TyG-BMI, triglyceride glucose-body mass index; TyG, triglyceride glucose; HOMA-IR, homeostatic model assessment of insulin resistance

In contrast to the non-periodontitis group, the periodontitis group exhibited several distinguishing features: They were notably older, had a larger WC, higher smoking rates, a greater proportion of males, increased prevalence of diabetes and hypertension, and elevated levels of TG, fasting glucose, fasting insulin, and all TyG-related indices. This group also showed lower educational levels, lower PIR, less frequent dental floss usage and a smaller number of permanent teeth.

For a comprehensive understanding of the distributional differences in each parameter, the participants were categorised into quartiles according to TyG-WHtR, TyG-WWI, TyG-WC, and TyG-BMI. Participants in the top quartile for TyG-WHtR, TyG-WWI, and TyG-WC exhibited an increased prevalence in periodontitis in comparison to the bottom quartile, and were more prone to possess lower educational and income levels, less frequent dental floss usage, a smaller number of permanent teeth, higher obesity levels or more recurrent overweight status, more frequent smoking assignments, and a greater prevalence of diabetes and hypertension (Table 2 and Supplementary Tables 1, 2, and 3).

**Table 2:** Baseline characteristics according to TyG-WHtR quartiles in NHANES 2009–2014

TyG-WHtR	Quartile 1	Quartile 2	Quartile 3	Quartile 4	*P* value
	(2.94–4.47)	(4.47–5.08)	(5.08–5.77)	(5.77–10.64)	
**Age (%)**					< 0.001
< 60	80.7	73.1	70.6	68.1	
≥ 60	19.3	26.9	29.4	31.9	
**Gender (%)**					0.001
Male	44.4	54.6	52.5	46.9	
Female	55.6	45.4	47.5	53.1	
**Race (%)**					< 0.001
Mexican American	4.0	7.0	11.2	10.9	
Other Hispanic	5.1	5.5	5.9	6.1	
Non-Hispanic White	71.2	69.5	67.4	69.9	
Non- Hispanic Black	10.7	9.2	9.8	9.1	
Other Races	9.0	8.8	5.7	4.1	
**Education level (%)**					< 0.001
Less than high school	11.1	14.5	17.5	21.6	
High school and above	88.9	85.5	82.5	78.4	
**PIR (%)**					< 0.001
< 1	9.9	9.3	10.6	15.1	
≥ 1	90.1	90.7	89.4	84.9	
**BMI (%)**					< 0.001
< 25	74.9	23.7	4.0	0.1	
≥ 25	25.1	76.3	96.0	99.9	
**Alcohol (%)**					< 0.001
No	16.1	17.3	19.4	26.2	
Yes	83.9	82.7	80.6	73.8	
**Smoke (%)**					0.024
No	60.6	58.8	54.4	54.1	
Yes	39.4	41.2	45.6	45.9	
**Physical activity (%)**					0.307
No	61.2	61.3	59.0	64.4	
Yes	38.8	38.7	41.0	35.6	
**Diabetes (%)**					< 0.001
No	97.1	91.3	82.6	61.2	
Yes	2.9	8.7	17.4	38.8	
**Hypertension (%)**					< 0.001
No	80.0	64.2	53.2	40.6	
Yes	20.0	35.8	46.8	59.4	
**Dental floss (%)**					0.001
No	25.0	26.8	29.0	33.6	
Yes	75.0	73.2	71.0	66.4	
**Dentition status (%)**					< 0.001
Non-functional	9.5	13.4	13.5	19.8	
Functional	90.5	86.6	86.5	80.2	
**Periodontitis (%)**					< 0.001
No	65.3	60.8	56.5	51.0	
Yes	34.7	39.2	43.5	49.0	

Categorical variables were presented as %, the *P*-value was derived using a weighted chi-square test

Abbreviations: PIR, income-to-poverty ratio; BMI, body mass index; TyG-WHtR, triglyceride glucose-waist to height ratio

### Associations between TyG-related obesity indices and periodontitis

Multivariable logistic regression models were employed to examine the links of TyG-WHtR, TyG-WWI, TyG-WC, and TyG-BMI with periodontitis (Supplementary Table 4). TyG-WHtR [odds ratios (OR) (95% confidence intervals (CI)) = 2.83 (1.58–5.10), *P* = 0.002], TyG-WWI [OR (95% CI) = 7.50 (3.06–18.34), *P* < 0.001], and TyG-WC [OR (95% CI) = 2.12 (1.23–3.64), *P* = 0.011] presented a strong positive correlation with periodontitis in the full adjustment model. Stronger positive correlations were observed in the highest quartile than those in the lowest quartile (Table 3). Specifically, TyG-WWI [OR (95% CI) = 1.72 (1.26–2.33), *P* = 0.001] and TyG-WC [OR (95% CI) = 1.50 (1.13–1.99), *P* = 0.009] exhibited a more pronounced positive correlation in model 3, indicating that the higher indices may be associated with an increased risk of periodontitis. Nevertheless, TyG-BMI did not present a discernible trend in association with the periodontitis in the preceding analysis.

**Table 3:** Associations between TyG-related obesity indices and periodontitis

	Odds ratio (95% confidence interval), *P* value					
	Model 1		Model 2		Model 3	
**TyG**-**WHtR**						
Quartile 1	Ref.		Ref.		Ref.	
Quartile 2	1.22 (1.01–1.46)	0.041	1.06 (0.87–1.29)	0.593	1.15 (0.90–1.46)	0.262
Quartile 3	1.45 (1.13–1.87)	0.005	1.22 (0.94–1.59)	0.143	1.30 (0.94–1.80)	0.127
Quartile 4	1.81 (1.40–2.35)	< 0.001	1.61 (1.23–2.10)	0.001	1.38 (0.97–1.98)	0.083
***P *** **for trend**	< 0.001		0.001		0.069	
**TyG**-**WWI**						
Quartile 1	Ref.		Ref.		Ref.	
Quartile 2	1.58 (1.26–1.98)	< 0.001	1.46 (1.15–1.87)	0.004	1.41 (1.10–1.80)	0.008
Quartile 3	1.71 (1.32–2.22)	< 0.001	1.48 (1.12–1.97)	0.009	1.30 (0.97–1.73)	0.073
Quartile 4	2.62 (1.99–3.43)	< 0.001	2.27 (1.69–3.06)	< 0.001	1.72 (1.26–2.33)	0.001
***P *** **for trend**	< 0.001		< 0.001		0.002	
**TyG**-**WC**						
Quartile 1	Ref.		Ref.		Ref.	
Quartile 2	1.37 (1.14–1.65)	0.002	1.19 (0.98–1.45)	0.080	1.38 (1.06–1.80)	0.022
Quartile 3	1.69 (1.36–2.09)	< 0.001	1.37 (1.09–1.73)	0.009	1.62 (1.22–2.15)	0.002
Quartile 4	1.84 (1.51–2.25)	< 0.001	1.53 (1.22–1.93)	0.001	1.50 (1.13–1.99)	0.009
***P *** **for trend**	< 0.001		0.001		0.017	
**TyG**-**BMI**						
Quartile 1	Ref.		Ref.		Ref.	
Quartile 2	1.00 (0.82–1.22)	0.991	0.85 (0.69–1.05)	0.138	0.93 (0.70–1.23)	0.603
Quartile 3	1.19 (0.97–1.47)	0.104	1.01 (0.81–1.25)	0.956	1.05 (0.71–1.57)	0.796
Quartile 4	1.36 (1.11–1.66)	0.005	1.25 (1.00-1.57)	0.062	1.07 (0.75–1.53)	0.710
***P *** **for trend**	0.002		0.024		0.353	

Model 1: Without adjustment

Model 2: Adjusted for age, gender, and race

Model 3: Adjusted for all potential confounders: age, gender, race, education, PIR, BMI, physical activity, alcohol intake, smoking, hypertension, diabetes, dental floss, and dentition status

Abbreviations: Ref., reference; TyG, triglyceride glucose; TyG-WHtR, triglyceride glucose-waist to height ratio; TyG-WWI, triglyceride glucose-weight-adjusted waist index; TyG-WC, triglyceride glucose-waist circumference; TyG-BMI, triglyceride glucose-body mass index

Supplementary Table 4 offers further insights by comparing the connections of both TyG-related obesity indices and traditional IR index such as homeostatic model assessment of insulin resistance (HOMA-IR) with periodontitis. The combined indices TyG-WHtR, TyG-WWI, and TyG-WC showed stronger associations with periodontitis, emphasising the potential significance of the complex correlation between IR, obesity, and periodontitis.

### Subgroup analyses

The study conducted an in-depth examination of the intricate relationships between the TyG-related obesity indices and periodontitis through subgroup analyses accounting for BMI, gender, age, diabetes, and hypertension (Table 4). Interestingly, a significant correlation between TyG-WHtR, TyG-WWI, or TyG-WC and periodontitis was observed for participants of < 60 years old, with BMI ≥ 25, and without diabetes. However, no significant interactions were observed between the TyG-related obesity indices and periodontitis (*P* > 0.05 for all interactions).

**Table 4:** Subgroup analyses for the associations of TyG-related obesity indices with periodontitis

	**OR (95% CI)**	***P *** **value**	***P *** **for interaction**
	**TyG-WHtR**		
**Age**			0.199
< 60	2.70 (1.59–4.57)	< 0.001	
≥ 60	1.55 (0.70–3.42)	0.282	
**Gender**			0.964
Male	2.39 (1.22–4.68)	0.011	
Female	2.34 (1.27–4.31)	0.006	
**BMI**			0.888
< 25	2.21 (0.70–7.02)	0.178	
≥ 25	2.42 (1.44–4.09)	0.001	
**Diabetes**			0.884
No	2.57 (1.49–4.43)	0.001	
Yes	2.37 (0.92–6.10)	0.074	
**Hypertension**			0.296
No	2.96 (1.60–5.45)	0.001	
Yes	1.85 (0.93–3.66)	0.077	
	**TyG**-**WWI**		
**Age**			0.196
< 60	6.78 (3.07-15.00)	< 0.001	
≥ 60	2.61 (0.73–9.25)	0.138	
**Gender**			0.311
Male	4.09 (1.64–10.21)	0.003	
Female	7.22 (3.09–16.91)	< 0.001	
**BMI**			0.618
< 25	4.06 (1.10-14.98)	0.035	
≥ 25	5.91 (2.71–12.89)	< 0.001	
**Diabetes**			0.752
No	5.30 (2.46–11.39)	< 0.001	
Yes	6.75 (1.74–26.13)	0.006	
**Hypertension**			0.173
No	7.76 (3.29–18.30)	< 0.001	
Yes	3.31 (1.23–8.93)	0.018	
	**TyG**-**WC**		
**Age**			0.314
< 60	1.95 (1.12–3.39)	0.018	
≥ 60	1.14 (0.47–2.78)	0.777	
**Gender**			0.445
Male	1.44 (0.72–2.85)	0.301	
Female	2.06 (1.09–3.89)	0.025	
**BMI**			0.595
< 25	1.29 (0.41–4.11)	0.664	
≥ 25	1.82 (1.09–3.04)	0.022	
**Diabetes**			0.820
No	1.74 (1.01–2.97)	0.045	
Yes	1.97 (0.76–5.08)	0.162	
**Hypertension**			0.712
No	1.88 (1.02–3.48)	0.044	
Yes	1.59 (0.81–3.14)	0.179	
	**TyG**-**BMI**		
**Age**			0.502
< 60	1.34 (0.85–2.10)	0.205	
≥ 60	1.00 (0.49–2.06)	1.000	
**Gender**			0.696
Male	1.37 (0.76–2.44)	0.295	
Female	1.17 (0.71–1.94)	0.535	
**BMI**			0.450
< 25	0.82 (0.27–2.49)	0.729	
≥ 25	1.30 (0.86–1.95)	0.214	
**Diabetes**			0.795
No	1.32 (0.85–2.06)	0.214	
Yes	1.18 (0.57–2.47)	0.656	
**Hypertension**			0.949
No	1.24 (0.73–2.13)	0.429	
Yes	1.27 (0.74–2.19)	0.381	

The model is not adjusted for the stratification variable itself

Abbreviations: OR, odds ratio; 95% CI, 95% confidence interval; BMI, body mass index; TyG-WHtR, triglyceride glucose-waist to height ratio; TyG-WWI, triglyceride glucose-weight-adjusted-waist index; TyG-WC, triglyceride glucose-waist circumference; TyG-BMI, triglyceride glucose-body mass index; TyG, triglyceride glucose

### Dose-response relationships based on restricted cubic spline (RCS)

**Fig. 2:**
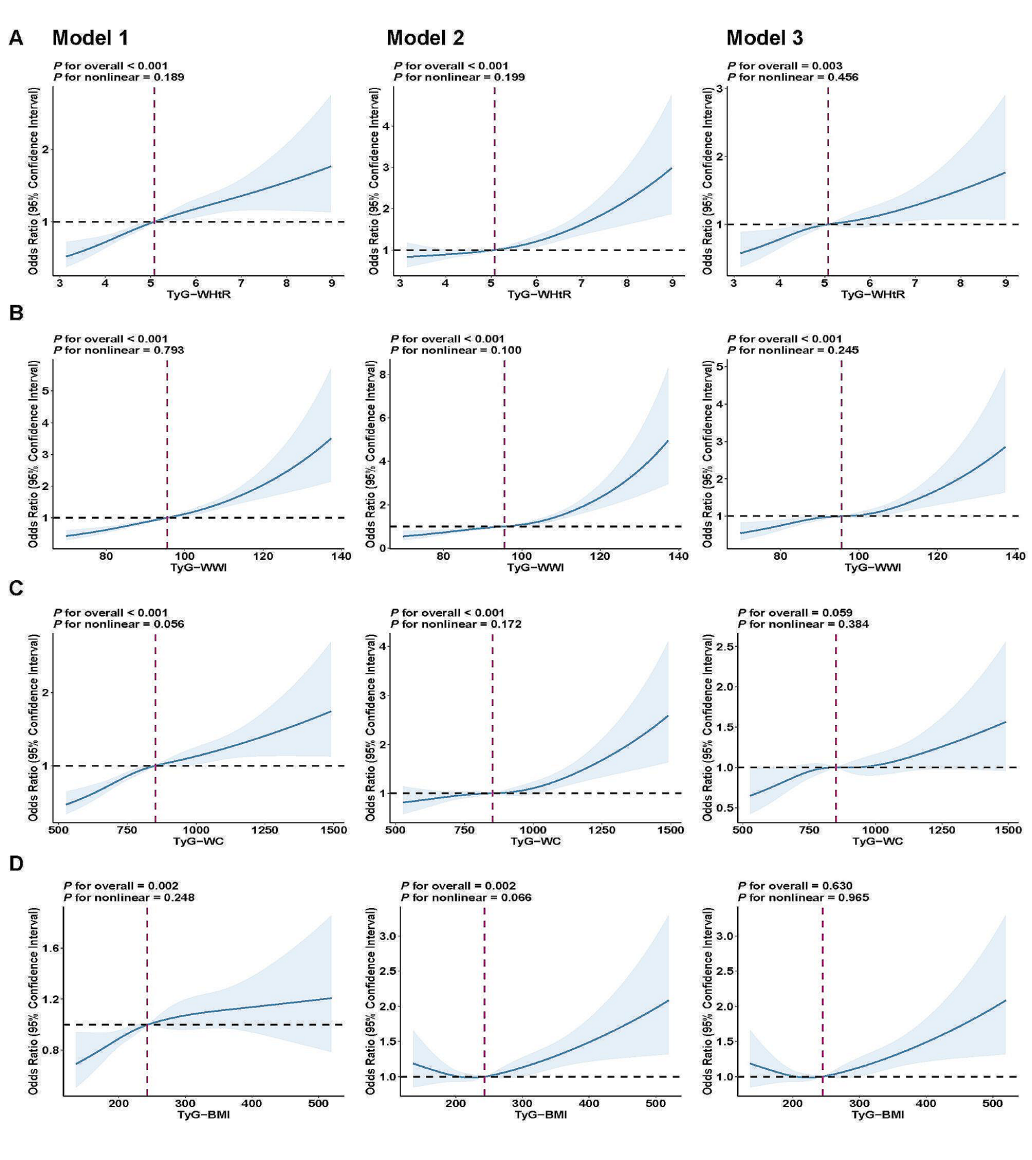
Restricted cubic spline (RCS) fitting for the associations of TyG-related obesity indices with periodontitis in different models. **(A)** RCS fitting for TyG-WHtR and periodontitis; **(B)** RCS fitting for TyG-WWI and periodontitis; **(C)** RCS fitting for TyG-WC and periodontitis; **(D)** RCS fitting for TyG-BMI and periodontitis

This study utilized RCS curves to flexibly model the variations in the data and explore the linearity of the correlations between TyG-WHtR, TyG-WWI, TyG-WC, or TyG-BMI and periodontitis. Figure 2 presents positive linear correlations *(P-nonlinear* > 0.05) between TyG-WHtR, TyG-WWI, and TyG-WC and periodontitis in the three adjustment models.

## Discussion

Throughout this national observational study, the correlation between IR, assessed by TyG-related obesity indices, and periodontitis was investigated utilizing the NHANES database for 2009–2014. The predominant discoveries revealed that TyG-WHtR, TyG-WWI, and TyG-WC, but not TyG-BMI, were positively correlated with the occurrence of periodontitis in the full adjustment model. Furthermore, participants in the top quartile had a higher risk of periodontitis relative to those in the bottom quartile, with a risk of 72% associated with TyG-WWI and 50% with TyG-WC. Subgroup analyses showed that strong relationships between TyG-WHtR, TyG-WWI, and TyG-WC and periodontitis were more likely to be observed among participants who were < 60 years of age, had a BMI ≥ 25, and no diabetes. These results indicated that recognizing the potential of TyG combined with obesity indices in epidemiological studies of periodontitis is essential for exploring the relationship between IR and this disease.

Previous studies have indicated that IR-associated metabolic syndrome is not only critical in the development and progression of diabetes but also increases the risk of developing periodontitis [22, 23]. The HOMA-IR, which is based on fasting glucose and insulin levels, is a classical IR index [24]. However, given the cost of insulin detection and the limitations of HOMA-IR in populations receiving insulin therapy, a more representative, efficient, and convenient index is still required. The TyG index has emerged as a comprehensive alternative in the estimation of IR due to its simplicity of calculation, sensitivity, and specificity [25]. Prior researches have shown that it provides comparable or better performance than HOMA-IR in evaluating IR [26–28]. Importantly, individuals with an increased level of IR usually present with obesity, making it a noteworthy driver of periodontitis occurrence [29]. The validity of the traditional anthropometric parameters, including WHtR, WC, and BMI, as well as the latest proposed WWI in predicting obesity has been confirmed [13]. Recently, the applicability of the TyG-related obesity indices has been widely investigated in chronic conditions such as cardiovascular disease [30, 31]. A national cohort study reported that integrating obesity metrics with TyG could better predictive metabolic syndrome risk than using these indices alone [32]. Furthermore, in the general population, TyG-WHtR, TyG-WC, and TyG-BMI were usually more robust in predicting IR compared to TyG alone [33]. Thus, combining obesity indices with TyG may more accurately reflect the correlation between IR and periodontitis.

Several studies have been conducted to support our discovery. According to Lee et al. [34], a significant association between the TyG index and periodontitis among Korean adults. Benguigui et al. [35] displayed a positive relationship between HOMA-IR and periodontitis, which was compatible with our findings. In addition, analysis of a cross-sectional study including 13,684 Koreans revealed a statistically significant elevation in the risk of periodontitis among those in the highest quartile for TyG-WHtR, TyG-WC, and TyG-BMI compared with those in the lowest quartile [36]. Notably, there was no correlation between TyG-BMI and periodontitis in the full adjustment model in this study, the reason is likely to be that the accuracy of using BMI to reflect obesity varies by disease and region [37, 38]. Hence, the superiority of TyG-related obesity metrics over single metrics remains controversial, and additional research is necessary to elucidate the different features of the parameters.

Although the precise pathophysiological relevance of the correlations between TyG-related obesity indices and periodontitis remains unclear, several factors may account for this relationship. The crosstalk between IR and oxidative stress as well as inflammation alters the host immune response. Mass inflammatory cytokine and mediator production causes abnormal insulin receptor signalling that ultimately impairs downstream metabolism [8]. Notably, these inflammatory mechanisms are closely correlated with periodontal bacterial lipopolysaccharide production in oral flora dysbiosis [39]. A recent investigation by Zeze et al. [40] showed that IR decreased the expression of vascular cell adhesion molecules, which exacerbated the inflammatory response in periodontal tissues by interfering insulin-mediated Forkhead Box O1 activity. Additionally, elevated levels of oxidative stress directly contribute to periodontal tissue destruction by inducing lipid metabolism abnormalities and others, protein structural alterations, and cell membrane disruption [41]. Furthermore, chronic inflammation induced by obesity not only affects insulin sensitivity but also promotes oral immune microenvironment disorders, ultimately exacerbating the severity of periodontitis [23, 42]. As a measure of obesity, BMI is commonly taken to describe overall obesity, while WC and WHtR are recognized as metrics of central obesity. Simultaneously, WWI is a new anthropometric parameter designed to represent central obesity regardless of body weight [43]. The location of fat distribution is known to has stronger implications for obesity-related risk than the amount of fat. For example, abdominal obesity is more closely associated with IR and metabolic dysfunction than peripheral obesity because it affects insulin metabolism by altering the release of fatty acids [44].

### Study strengths and limitations

There are several advantages to this study. Notably, it provided valuable evidence highlighting the positive relationships between TyG-related obesity indices and periodontitis. As the data originated from the NHANES database, the dataset was characterised by a substantial sample size and national representativeness. Furthermore, these analyses accounted for confounding variables associated with periodontitis, thus reinforcing the reliability of findings. However, certain limitations warrant consideration. First, the observational study design limited the ability to explore the causative correlations between TyG-related obesity indices and periodontitis. Second, despite efforts to adjust for various confounding factors, fully eliminating the potential impact of other confounding variables remains to be a challenge. Finally, this research focused on the US population, which may restrict the broader applicability of the findings to the global population. Future studies should consider larger and more diverse cohorts to determine the most effective TyG-related obesity indices for predicting periodontitis and to establish universally applicable threshold values.

## Conclusions

The study observed a strong association between TyG-WHtR, TyG-WWI, or TyG-WC and periodontitis in the American population. This underscores the potential of TyG-related obesity indices for identifying periodontitis risk, and emphasises the importance of fat distribution and insulin resistance in the pathogenesis of periodontitis. This study offers valuable perspectives for risk stratification, early intervention strategies, and cost-effective screening methods in high-risk populations.

### Electronic supplementary material

Below is the link to the electronic supplementary material.

Supplementary Material 1

Supplementary Material 2

Supplementary Material 3

Supplementary Material 4

## Data Availability

The NHANES data that support the findings of this study are openly available at https://www.cdc.gov/nchs/nhanes/index.htm.

## Abbreviations

NHANES: National Health and Nutrition Examination Survey
TyG: Triglyceride glucose
WHtR: Waist-to-height ratio
WWI: Weight-adjusted-waist index
BMI: Body mass index
WC: Waist circumference
RCS: Restricted cubic spline
IR: Insulin resistance
TG: Triglyceride
PIR: Income-to-poverty ratio
