# Supplementary material for: Associations between different triglyceride glucose index-related obesity indices and periodontitis: results from NHANES 2009–2014
Source: Lipids Health Dis. 2024 Jul 5;23:213. doi: 10.1186/s12944-024-02192-z (PMC11225363; doi:10.1186/s12944-024-02192-z)
Supplement: Supplementary file 1 — Supplementary Material 1 [file 12944_2024_2192_MOESM1_ESM.docx]

**Supplementary Table 1. Baseline characteristics according to TyG**-**WWI quartiles in NHANES 2009**-**2014**

| **TyG**-**WWI** | **Quartile 1** | **Quartile 2** | **Quartile 3** | **Quartile 4** | ***P* value** |
| --- | --- | --- | --- | --- | --- |
|  | **(63.50**-**87.97)** | **(87.97**-**95.41)** | **(95.41**-**103.07)** | **(103.08**-**149.14)** |  |
| **Age (%)** |  |  |  |  | <0.001 |
| < 60 | 84.7 | 77.6 | 70.0 | 58.7 |  |
| ≥ 60 | 15.3 | 22.4 | 30.0 | 41.3 |  |
| **Gender (%)** |  |  |  |  | 0.024 |
| Male | 44.6 | 53.6 | 51.9 | 48.4 |  |
| Female | 55.4 | 46.4 | 48.1 | 51.6 |  |
| **Race (%)** |  |  |  |  | <0.001 |
| Mexican American | 4.4 | 8.2 | 10.4 | 10.1 |  |
| Other Hispanic | 5.0 | 4.7 | 6.4 | 6.5 |  |
| Non-Hispanic White | 69.6 | 70.0 | 66.8 | 71.7 |  |
| Non- Hispanic Black | 14.4 | 10.3 | 7.9 | 5.5 |  |
| Other Races | 6.5 | 6.9 | 8.5 | 6.1 |  |
| **Education level (%)** |  |  |  |  | <0.001 |
| Less than high school | 9.5 | 14.9 | 19.1 | 21.6 |  |
| High school and above | 90.5 | 85.1 | 80.9 | 78.4 |  |
| **PIR (%)** |  |  |  |  | 0.013 |
| < 1 | 9.2 | 10.1 | 12.4 | 13.4 |  |
| ≥ 1 | 90.8 | 89.9 | 87.6 | 86.6 |  |
| **BMI (%)** |  |  |  |  | <0.001 |
| < 25 | 53.4 | 26.5 | 15.2 | 7.9 |  |
| ≥ 25 | 46.6 | 73.5 | 84.8 | 92.1 |  |
| **Alcohol (%)** |  |  |  |  | <0.001 |
| No | 17.0 | 16.0 | 21.4 | 24.9 |  |
| Yes | 83.0 | 84.0 | 78.6 | 75.1 |  |
| **Smoke (%)** |  |  |  |  | 0.001 |
| No | 63.9 | 57.2 | 54.8 | 51.4 |  |
| Yes | 36.1 | 42.8 | 45.2 | 48.6 |  |
| **Physical activity** |  |  |  |  | 0.588 |
| No | 61.7 | 59.8 | 60.5 | 63.9 |  |
| Yes | 38.3 | 40.2 | 39.5 | 36.1 |  |
| **Diabetes (%)** |  |  |  |  | <0.001 |
| No | 97.0 | 92.3 | 83.8 | 58.0 |  |
| Yes | 3.0 | 7.7 | 16.2 | 42.0 |  |
| **Hypertension (%)** |  |  |  |  | <0.001 |
| No | 79.0 | 63.2 | 54.2 | 40.4 |  |
| Yes | 21.0 | 36.8 | 45.8 | 59.6 |  |
| **Dental floss (%)** |  |  |  |  | 0.031 |
| No | 25.1 | 28.7 | 28.3 | 32.4 |  |
| Yes | 74.9 | 71.3 | 71.7 | 67.6 |  |
| **Dentition status (%)** |  |  |  |  | <0.001 |
| Non-functional | 7.7 | 11.2 | 16.5 | 21.5 |  |
| Functional | 92.3 | 88.8 | 83.5 | 78.5 |  |
| **Periodontitis (%)** |  |  |  |  | <0.001 |
| No | 69.6 | 59.2 | 57.2 | 46.7 |  |
| Yes | 30.4 | 40.8 | 42.8 | 53.3 |  |

Categorical variables were presented as %, the *P*-value was derived using a weighted chi-square test.

Abbreviations: PIR, income-to-poverty ratio; BMI, body mass index; TyG-WWI, triglyceride glucose-weight-adjusted-waist index.
