# Supplementary material for: Associations between different triglyceride glucose index-related obesity indices and periodontitis: results from NHANES 2009–2014
Source: Lipids Health Dis. 2024 Jul 5;23:213. doi: 10.1186/s12944-024-02192-z (PMC11225363; doi:10.1186/s12944-024-02192-z)
Supplement: Supplementary file 2 — Supplementary Material 2 [file 12944_2024_2192_MOESM2_ESM.docx]

**Supplementary Table 2. Baseline characteristics according to TyG**-**WC quartiles in NHANES 2009**-**2014**

| **TyG**-**WC** | **Quartile 1** | **Quartile 2** | **Quartile 3** | **Quartile 4** | ***P* value** |
| --- | --- | --- | --- | --- | --- |
|  | **(476.39**-**745.86)** | **(745.86**-**849.61)** | **(849.73**-**967.46)** | **(967.51**-**1646.76)** |  |
| **Age (%)** |  |  |  |  | 0.001 |
| < 60 | 78.7 | 73.3 | 71.0 | 70.3 |  |
| ≥ 60 | 21.3 | 26.7 | 29.0 | 29.7 |  |
| **Gender (%)** |  |  |  |  | <0.001 |
| Male | 30.1 | 48.9 | 58.5 | 60.2 |  |
| Female | 69.9 | 51.1 | 41.5 | 39.8 |  |
| **Race (%)** |  |  |  |  | <0.001 |
| Mexican American | 5.9 | 7.6 | 10.1 | 8.9 |  |
| Other Hispanic | 6.0 | 5.7 | 5.6 | 5.2 |  |
| Non-Hispanic White | 67.1 | 68.2 | 69.4 | 73.3 |  |
| Non- Hispanic Black | 10.0 | 10.1 | 9.5 | 9.2 |  |
| Other Races | 10.9 | 8.4 | 5.3 | 3.3 |  |
| **Education level (%)** |  |  |  |  | 0.001 |
| Less than high school | 12.8 | 14.9 | 18.0 | 18.4 |  |
| High school and above | 87.2 | 85.1 | 82.0 | 81.6 |  |
| **PIR (%)** |  |  |  |  | 0.041 |
| < 1 | 11.0 | 9.7 | 10.7 | 13.2 |  |
| ≥ 1 | 89.0 | 90.3 | 89.3 | 86.8 |  |
| **BMI (%)** |  |  |  |  | <0.001 |
| < 25 | 77.7 | 25.7 | 4.8 | 0.2 |  |
| ≥ 25 | 22.3 | 74.3 | 85.2 | 99.8 |  |
| **Alcohol (%)** |  |  |  |  | 0.013 |
| No | 19.7 | 18.2 | 17.5 | 23.0 |  |
| Yes | 80.3 | 81.8 | 82.5 | 77.0 |  |
| **Smoke (%)** |  |  |  |  | 0.001 |
| No | 62.6 | 59.0 | 54.8 | 52.2 |  |
| Yes | 37.4 | 41.0 | 45.2 | 47.8 |  |
| **Physical activity** |  |  |  |  | 0.593 |
| No | 61.3 | 63.5 | 59.5 | 61.4 |  |
| Yes | 38.7 | 36.5 | 40.5 | 38.6 |  |
| **Diabetes (%)** |  |  |  |  | <0.001 |
| No | 96.8 | 90.0 | 85.2 | 62.6 |  |
| Yes | 3.2 | 10.0 | 14.8 | 37.4 |  |
| **Hypertension (%)** |  |  |  |  | <0.001 |
| No | 78.8 | 65.4 | 54.6 | 41.9 |  |
| Yes | 21.2 | 34.6 | 45.4 | 58.1 |  |
| **Dental floss (%)** |  |  |  |  | <0.001 |
| No | 22.4 | 27.6 | 29.9 | 33.9 |  |
| Yes | 77.6 | 72.4 | 70.1 | 66.1 |  |
| **Dentition status (%)** |  |  |  |  | <0.001 |
| Non-functional | 10.1 | 14.1 | 13.7 | 17.8 |  |
| Functional | 89.9 | 85.9 | 86.3 | 82.2 |  |
| **Periodontitis (%)** |  |  |  |  | <0.001 |
| No | 67.2 | 59.9 | 54.9 | 52.7 |  |
| Yes | 32.8 | 40.1 | 45.1 | 47.3 |  |

Categorical variables were presented as %, the *P*-value was derived using a weighted chi-square test.

Abbreviations: PIR, income-to-poverty ratio; BMI, body mass index; TyG-WC, triglyceride glucose-waist circumference.
