# Supplementary material for: Associations between different triglyceride glucose index-related obesity indices and periodontitis: results from NHANES 2009–2014
Source: Lipids Health Dis. 2024 Jul 5;23:213. doi: 10.1186/s12944-024-02192-z (PMC11225363; doi:10.1186/s12944-024-02192-z)
Supplement: Supplementary file 3 — Supplementary Material 3 [file 12944_2024_2192_MOESM3_ESM.docx]

**Supplementary Table 3. Baseline characteristics according to TyG**-**BMI quartiles in NHANES 2009-2014**

| **TyG**-**BMI** | **Quartile 1** | **Quartile 2** | **Quartile 3** | **Quartile 4** | ***P* value** |
| --- | --- | --- | --- | --- | --- |
|  | **(117.18**-**208.55)** | **(208.58-244.30)** | **(244.31-287.18)** | **(287.20-679.46)** |  |
| **Age (%)** |  |  |  |  | 0.753 |
| < 60 | 73.7 | 72.2 | 72.7 | 74.6 |  |
| ≥ 60 | 26.3 | 27.8 | 27.3 | 25.4 |  |
| **Gender (%)** |  |  |  |  | <0.001 |
| Male | 39.1 | 55.9 | 54.1 | 49.2 |  |
| Female | 60.9 | 44.1 | 45.9 | 50.8 |  |
| **Race (%)** |  |  |  |  | <0.001 |
| Mexican American | 4.4 | 7.4 | 11.1 | 9.8 |  |
| Other Hispanic | 4.8 | 5.6 | 6.8 | 5.4 |  |
| Non-Hispanic White | 71.5 | 69.9 | 67.0 | 69.7 |  |
| Non- Hispanic Black | 8.0 | 9.1 | 10.3 | 11.4 |  |
| Other Races | 11.3 | 8.0 | 4.8 | 3.7 |  |
| **Education level (%)** |  |  |  |  | <0.001 |
| Less than high school | 13.3 | 13.8 | 18.0 | 19.3 |  |
| High school and above | 86.7 | 86.2 | 82.0 | 80.7 |  |
| **PIR (%)** |  |  |  |  | 0.014 |
| < 1 | 10.4 | 9.9 | 10.8 | 13.6 |  |
| ≥ 1 | 89.6 | 90.1 | 89.2 | 86.4 |  |
| **BMI (%)** |  |  |  |  | <0.001 |
| < 25 | 88.8 | 17.2 | 0.2 | 0.0 |  |
| ≥ 25 | 11.2 | 82.8 | 99.8 | 100.0 |  |
| **Alcohol (%)** |  |  |  |  | <0.001 |
| No | 17.6 | 17.1 | 18.9 | 25.0 |  |
| Yes | 82.4 | 82.9 | 81.1 | 75.0 |  |
| **Smoke (%)** |  |  |  |  | 0.586 |
| No | 58.5 | 57.5 | 57.2 | 55.1 |  |
| Yes | 41.5 | 42.5 | 42.8 | 44.9 |  |
| **Physical activity** |  |  |  |  | 0.440 |
| No | 61.7 | 63.0 | 58.6 | 62.3 |  |
| Yes | 38.3 | 37.0 | 41.4 | 37.7 |  |
| **Diabetes (%)** |  |  |  |  | <0.001 |
| No | 95.5 | 89.8 | 83.8 | 64.9 |  |
| Yes | 4.5 | 10.2 | 16.2 | 35.1 |  |
| **Hypertension (%)** |  |  |  |  | <0.001 |
| No | 76.9 | 64.3 | 55.7 | 42.9 |  |
| Yes | 23.1 | 35.7 | 44.3 | 57.1 |  |
| **Dental floss (%)** |  |  |  |  | <0.001 |
| No | 24.4 | 26.5 | 28.5 | 34.6 |  |
| Yes | 75.6 | 73.5 | 71.5 | 65.4 |  |
| **Dentition status (%)** |  |  |  |  | 0.026 |
| Non-functional | 12.3 | 13.3 | 13.5 | 16.7 |  |
| Functional | 87.7 | 86.7 | 86.5 | 83.3 |  |
| **Periodontitis (%)** |  |  |  |  | 0.007 |
| No | 61.5 | 61.5 | 57.2 | 54.0 |  |
| Yes | 38.5 | 38.5 | 42.8 | 46.0 |  |

Categorical variables were presented as %, the *P*-value was derived using a weighted chi-square test.

Abbreviations: income-to-poverty ratio; BMI, body mass index; PIR, TyG-BMI, triglyceride glucose-body mass index.
