# Supplementary material for: Associations between different triglyceride glucose index-related obesity indices and periodontitis: results from NHANES 2009–2014
Source: Lipids Health Dis. 2024 Jul 5;23:213. doi: 10.1186/s12944-024-02192-z (PMC11225363; doi:10.1186/s12944-024-02192-z)
Supplement: Supplementary file 4 — Supplementary Material 4 [file 12944_2024_2192_MOESM4_ESM.docx]

**Supplementary Table 4. Multivariable logistic regression analysis of TyG**-**related indices, TyG, and HOMA-IR with periodontitis.**

|  | **Odds ratio (95% confidence interval), *P* value** | | | | | |  |
| --- | --- | --- | --- | --- | --- | --- | --- |
|  | **Model 1** | | **Model 2** | | **Model 3** | | |
| TyG-WHtR | 3.81 (2.44-5.93) | <0.001 | 3.15 (2.00-4.95) | <0.001 | 2.83 (1.58-5.10) | 0.002 | |
| TyG-WWI | 24.65 (11.73-51.80) | <0.001 | 16.74 (7.50-37.37) | <0.001 | 7.50 (3.06-18.34) | <0.001 | |
| TyG-WC | 3.42 (2.35-4.98) | <0.001 | 2.55 (1.67-3.89) | <0.001 | 2.12 (1.23-3.64) | 0.011 | |
| TyG-BMI | 1.71 (1.25-2.33) | 0.002 | 1.57 (1.13-2.18) | 0.010 | 1.38 (0.87-2.18) | 0.184 | |
| TyG | 1.46 (1.29-1.65) | <0.001 | 1.37 (1.20-1.56) | <0.001 | 1.15 (1.00-1.34) | 0.057 | |
| HOMA-IR | 1.39 (1.27-1.53) | <0.001 | 1.29 (1.17-1.43) | <0.001 | 1.24 (1.11-1.39) | 0.001 | |

Model 1: Without adjustment.

Model 2: Adjusted for age, gender, and race.

Model 3: Adjusted for all potential confounders: age, gender, race, education, PIR, BMI, physical activity, alcohol intake, smoking, hypertension, diabetes, dental floss, and dentition status.

Abbreviations: TyG-WWI, triglyceride glucose-weight-adjusted waist index; TyG-WHtR, triglyceride glucose-waist to height ratio; TyG-WC, triglyceride glucose-waist circumference; TyG-BMI, triglyceride glucose-body mass index; HOMA-IR, homeostatic model assessment of insulin resistance; TyG, triglyceride glucose
